# Evolution and study of a copycat effect in intimate partner homicides: A lesson from Spanish femicides

**DOI:** 10.1371/journal.pone.0217914

**Published:** 2019-06-06

**Authors:** José L. Torrecilla, Lara Quijano-Sánchez, Federico Liberatore, Juan J. López-Ossorio, José L. González-Álvarez

**Affiliations:** 1 UC3M-BS Institute of Financial Big Data, Universidad Carlos III de Madrid, Madrid, Spain; 2 Department of Mathematics, Universidad Autonóma de Madrid, Madrid, Spain; 3 Department of Computer Science, Universidad Autonóma de Madrid, Madrid, Spain; 4 Department of Statistics and Operational Research, Faculty of Mathematics, Universidad Complutense de Madrid, Madrid, Spain; 5 Gabinete de Coordinación y Estudios, Secretaría de Estado de Seguridad, Ministerio del Interior, Madrid, Spain; Anglia Ruskin University, UNITED KINGDOM

## Abstract

**Objectives:**

This paper focuses on the issue of intimate partner violence and, specifically, on the distribution of femicides over time and the existence of copycat effects. This is the subject of an ongoing debate often triggered by the social alarm following multiple intimate partner homicides (IPHs) occurring in a short span of time. The aim of this research is to study the evolution of IPHs and provide a far-reaching answer by rigorously analyzing and searching for patterns in data on femicides.

**Methods:**

The study analyzes an official dataset, provided by the system VioGén of the *Secretaría de Estado de Seguridad* (Spanish State Secretariat for Security), including all the femicides occurred in Spain in 2007-2017. A statistical methodology to identify temporal interdependencies in count time series is proposed and applied to the dataset. The same methodology can be applied to other contexts.

**Results:**

There has been a decreasing trend in the number of femicides per year. No interdependencies among the temporal distribution of femicides are observed. Therefore, according to data, the existence of copycat effect in femicides cannot be claimed.

**Conclusions:**

Around 2011 there was a clear change in the average number of femicides which has not picked up. Results allow for an informed answer to the debate on copycat effect in Spanish femicides. The planning of femicides prevention activities should not be a reaction to a perceived increase in their occurrence. As a copycat effect is not detected in the studied time period, there is no evidence supporting the need to censor media reports on femicides.

## Introduction

Violence against women represents a global health problem of epidemic proportions [1]. This disturbing reality has been described by United Nations [2] as one of the most usual violations of human rights. Among its many manifestations, intimate partner violence (IPV) represents physical violence, sexual violence, stalking or psychological aggression committed by a current or former intimate partner. To date, it is estimated that 35 per cent of women worldwide have experienced IPV [3, 4]. This alarming data poses gender violence in the spotlight of public debate [5, 6]. The World Health Organization (WHO) has declared that the prevention of gender violence is a priority that requires an exhaustive knowledge of its scale [7]. Among the many recommendations that the WHO has published, a primary role is assigned to understanding and recognising the different factors that promote this type of violence to design and invest in prevention strategies. In this paper we focus on the most extreme manifestation of IPVs, Intimate Partner Homicides (IPHs), also known as femicides.

This type of events, that reflect a very serious and disturbing reality, has been widely studied [8–13]. Day to day, the news and the press raise alarms in the population that claims for immediate action. In particular, when multiple IPHs happen within a short period of time suggesting a surge in murders, the urge to react and adopt preventing measures is more critical. The population expects governments to intervene and install public measures that work toward the protection of citizens. However, these actions should be informed and based on scientific facts, rather than popular beliefs or political pressure, to be as effective as possible and ensuring that resources are invested where they are most needed. In this regard, ĭt is essential to avoid falling into misleading conclusions produced by incorrect data processing or spurious correlations. Some sources of misinterpretation have been considered in works like [14].

For these and other reasons, the need to prevent or redesign how IPHs are reported in the media is widely debated, as well as the potential adoption of additional public measures [15, 16]. In this context, a key point is to determine the existence or not of a copycat effect in this type of crimes, derived by potential murderers finding out about similar cases through, for example, the appearance of news reporting IPHs.

In this paper, we study the evolution of IPHs in Spain from 2007 to 2017 and try to answer the question of whether there are changes in the distribution of femicides in time or not, due to the existence of a copycat effect or other causes. For this, we use common, contrasted and easily interpretable statistical tools with the largest sample considered to date in this type of studies: all IPHs officially studied and registered in Spain since the implantation of the current system, that is, 655 IPHs from the 1st of January of 2007 to the 31 of December of 2017. The analyzed data comes from the VioGén system [17] of the *Secretaría de Estado de Seguridad del Ministerio del Interior* (Spanish State Secretariat for Security of the Ministry of Interior) (SES), that is, the official source of information for this kind of events in Spain. This allows to delve into the preliminary results provided so far in the literature and give a more conclusive and informed answer to the questions about the evolution of femicides in Spain and the existence of a copycat effect in IPHs. Thus, guiding future research and investments in prevention and predictive factors. In fact, the current study improves on the previous researches (please refer to the Background section) in the following ways: (i) data are obtained from official sources (SES); (ii) data includes all the femicides in Spain comprised between 2007 and 2017 and, therefore, provides a wider time horizon and covers the whole national territory; (iii) data has not been contaminated by considering events other than consumed IPHs nor by mixing data sources; (iv) the statistical analysis is rigorous and addresses the hypothesis of copycat effect from a global perspective without relying on strong assumptions.

The results show clear changes in the volume and distribution of femicides throughout the historical series. More concretely, our tests show a significantly decreasing average number of femicides in recent years compared to those in the first years of the sample. Interestingly, the appearance of change-points joint with differences in some specific years, such as 2009, suggests the influence of external factors in this type of events and motivates further analysis.

In addition, our research has not revealed evidence in favor of the existence of a copycat effect. While we have identified a global decreasing trend, which rules out the random distribution of the events, our tests support the null hypothesis of a random distribution when the time series is split according to the change-point. Also, significant dependencies among consecutive events or inter-arrival times have not been appreciated. Moreover, a simulation study generating time series with and without copycat effect shows that our data are more similar to the latter than the former. The research is completed with some complementary tests and standard descriptive tools.

In the following section we summarize relevant background information on IPHs, including a literature review on the subject. Next, we describe the data and sources used in this analysis and the modeling and statistical methods considered. We then present the results obtained, followed by a discussion and insights for future research.

## Background

The copycat effect is defined in the specialized literature as a criminal act that is modelled or inspired by a previous crime that has been reported in the media or described in fiction [18, 19].

Despite the importance of the implications derived from the potential existence of an imitation effect on criminal behavior in both hetero-directed and self-directed violence, the empirical research on this topic is very scarce and there is controversy about the existence of this effect in most cases. Even the concept used poses terminological problems that make it difficult to systematically review the phenomenon [13, 20].

Most studies on the subject have focused on this effect in suicides, a self-directed violent behavior that shares some elements with other violent behaviors. The idea of a possible copycat effect in suicides due to imitation has its origins in the so-called “Werther effect” [21–23], i.e., a person attempts suicide to emulate another suicide that he/she learns about either from local knowledge, or accounts of depictions in the media. Historically, most of the evidence found focuses on suicide cases of young people following high-impact media events describing the suicide of very popular personalities [24]. A recent large-scale study analyzed this phenomenon in Japan between 1989 and 2010, with a sample of 8,035 suicides, concluding that media information on celebrity suicide (109 in total, among which were also politicians and well-known businesspersons) immediately increased suicides by a 5% on average and remained in effect for about 10 days after publication [25]. Other works [26–31] also support the existence of a copycat effect when it comes to suicide and present the existence or necessity of standards in their media coverage.

Related to our specific domain, IPHs, the first question that can be asked is if the results on suicide imitation can be extrapolated to hetero-directed violent behaviors. Helfgott [19] explains that the influence of media on criminal behavior depends on each individual. In Spain, a review of the empirical evidence of the influence of the media on aggressive behavior draws a complex scenario. Igartua [32] shows moderate effects on some violent behaviors, especially sexualized ones, through explanatory mechanisms. Research indicates that this influence is complex and includes the analysis of individual, situational, media, social, and cultural characteristics, among others. More recent works are trying to study the existence or not of these effects in other types of violent acts or crimes such as mass killings [33–36], robberies [37], terrorist acts [38, 39] or shootings [40, 41].

Within this framework, IPHs deserve special consideration because of their gravity and the media impact they have on societies. The existence of a common belief that there is a copycat effect in femicides resulting from the appearance of news of spurts of murders, along with studies that show that the knowledge of incidences in similar crimes is an enhancer in other types of violent acts, constitute a sound motivation to study the potential copycat effect in femicides.

The topic of copycat effect in IPHs has been addressed in both the academic literature [42–44] and the media [45]. Lorente [44] points out that “the very realization of a violent reality can act as reinforcement for many aggressors and as fuel for the fear that lives inside the threatened women.” Hence, according to the author, men think of homicide as a last resort when the mechanisms of control over the partner do not work and when they see in media models with which they identify and that reinforce their homicidal intention. Following this influence hypothesis, Vives-Cases *et al*. [43] analyze 340 homicides registered in Spain during the period 2003 and 2007, concluding that the presence of IPHs in television news increased the possibility of these events by 32% to 42%, noting that the results are consistent with studies of this effect in the case of suicides. However, this pioneer research presents some limitations, mostly due to the quality of the data used. In fact, the authors rely on unofficial and incomplete data (obtained from the news), and report an imperfect agreement of 89% between their collected data and official sources (i.e., Spanish Home Office). Also, the authors recognize that their study may have been affected by difficulties in accessing quantitative information about cases of death by Intimate Partner Violence (IPV). Other researches show similar problems. Marzabal Manresa [42] includes in her study only 30 femicides occurred in Barcelona between 2004 and 2009 to understand the effect on the likelihood of IPHs in the 10 days following the appearance of news of femicides in the media. Statistically, the dataset cannot be considered representative. Also, the study presents a methodological flaw as the author does not include in the analysis all the days that do not present neither an IPH nor news on the topic. Therefore, her conclusion that news reports on femicides increase their likelihood in the following days is biased and cannot be trusted. A different outcome is obtained by Teruelo [14] that states that no significant copycat effect can be detected in IPHs and deduces that the distribution of femicides is stationary over time. However, the author considers only six years of data and the study is contaminated by including reported tentative IPHs. In the paper it is not clear how these attempts are defined (they do not come from an official source) and their inclusion can introduce a source of uncertainty that should be taken into account when conducting the analysis and interpreting the results.

## Materials and methods

The analyses described in this section have been carried out using the software package R version 3.4.3 [46] and the following libraries: portes, tseries, snpar, AER, energy, MASS, dunn.test, forecast, change-point and goftest.

### Data

This research has been carried out in collaboration with SES, thus providing a thorough and reliable dataset about femicides in Spain. Note that the terms “IPH” and “femicide” are used interchangeably throughout this text. The dataset is comprised of the dates of all the femicides occurred in Spain from 2007 to 2017 (inclusive) and it has been obtained from the VioGén system [17] that includes all official reports of the Security Agencies of the country, i.e., *Policía Nacional* (Spanish Nacional Police Corps), *Guardia Civil* (Civil Guard), *Ertzaintza* (Police Force for the Basque Country), *Mossos d’Esquadra* (Police Force of Catalonia), and *Policía Foral* (Police Force of Navarra). This list of dates, does not reflect or identify neither the victim nor the murderer, is completely anonymized and of public knowledge. The Committee for Ethical Research of the Universidad Autonoma de Madrid determined that ethical review and participant consent were not necessary for this study. Researchers interested in the data can access it by request to the Servicio Central de Violencia de Género. The *Secretaría de Estado de Seguridad del Ministerio del Interior* (Spanish State Secretariat for Security of the Ministry of Interior) gives full consent on the publication of this research, i.e. the methodology, results, insights and data used to develop it.

Next, we give a thorough presentation of the methodology applied to confirm the following hypotheses: 1) There is a decreasing trend in the number of femicides per year; 2) There is no observed copycat effect in femicides.

### Modeling and methods: Evolution and trends in Spanish femicides

First of all, we determine that the distribution of femicides is not uniform along time as concluded in [14]. We show that there are differences throughout the historical series and we try to shed some light on the structure and trends.

The uniformity is contrasted with three standard test for goodness of fit that focus on different characteristics of the probability distribution: Kolmogorov-Smirnov (*KS*), Cramer von Mises (CVM) and Anderson-Darling (AD). To confer robustness we complement the outlined methodology with a runs test for the binarized data series [47] that searches for randomness by examining the sequences of similar responses. In all of these tests, the null hypothesis is the data are uniformly distributed. In fact, if the data can be assumed to be generated by a uniform distribution, then we can conclude that it is completely random in nature and there is no trend and copycat effect.

Besides, we also check if there are significant differences in the volume of murders in different years, months or days of the week by running a Dunn’s multiple comparison test, that is a post hoc non parametric test. The null hypothesis for the test is that there is no difference between groups (groups can be of different sizes). Dunn’s test can be a very conservative test, especially for larger numbers of comparisons. Therefore, differences detected by this test are highly significant.

Once the uniformity hypothesis of the complete series is discarded, we study its temporal structure. For this we look at the intensity function λ(*t*), which describes how the average number of femicides changes over time (λ(*t*) will be further explained in next section). This function can be approximated by any smoothing technique applied to the raw data. Here we estimate λ(*t*) by standard moving averages (MA) and study the existence of change-points and its trend by means of a Cox-Stuart test. This is also a conservative test, which implies that, if found, then the trend is clear. Also, the change-point analysis [48] on λ(*t*) provides another strong argument against the complete uniformity of the distribution of the events over time. As later detailed in the results section, all analyses carried out show evidences of an important change around the end of 2011 with clear differences in the behavior of the series before and after this point. We show the results for the most significant change-point (identified using the “At Most One Change,” AMOC method) with asymptotic penalty values and a 95% confidence.

### Modeling and methods: Copycat effect

This section describes the methodology used to ascertain the existence of a copycat effect in the data, that is, the probability of a femicide is higher in the days following an event. The literature on the subject mostly focused on two approaches.

The first approach relies on the use of classical statistical tools and tests. In this sense the adjustment of the events to Poisson processes implies the uniformity of the distribution of occurrences over time, that is, the absence of a copycat effect. Our study follows this philosophy. Previous contributions in the context of suicides and mass killings base their methodology on the use of the Poisson distribution and/or the Poisson process. This is the case of works like [26, 29, 36, 49]. Liesenfeld *et al*. [49] make use of the Poisson distribution to condition the behavior of certain variables. Cheng *et al*. [26] model suicide data as a non-homogeneous Poisson process. Ji *et al*. [29] use a Poisson time series auto-regression model to study suicide copycat effect in South Korea. The sparsity in our data does not allow the application of this exact methodology. In fact, their dataset is comprised of 34,237 events occurred in three years, while ours involves only 655 events in 11 years. Finally, King and Jacobson [36] fit data relative to mass killings in the United States to a Poisson process to determine whether their distribution is random. Due to the differences in the domain and the data distribution, we complement the methodology presented by these authors by including a simulation study. To the best of the authors knowledge, this is the first research that applies the proposed modeling strategy to the context of femicides.

**Fig 1:**
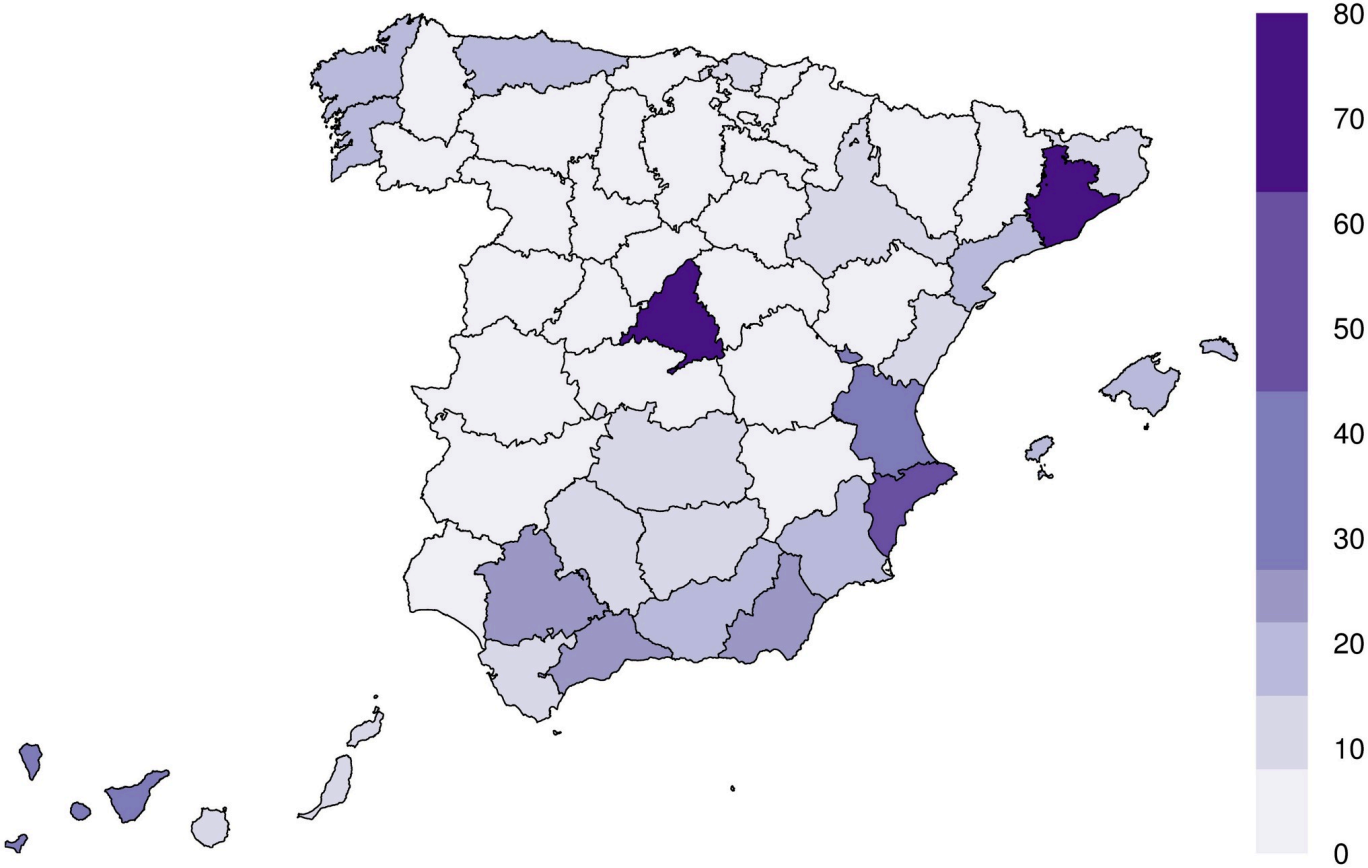
Geographical occurrence of femicides in Spain. Work derived from BDLJE May 2018 CC-BY 4.0 scne.es.

The second approach, used especially in the field of criminology, is based on the use of spatio-temporal models and self-exciting processes [34, 40]. Unfortunately, these models rely on specific conditions of data density (quantity and distribution) to correctly estimate their parameters, which is why they are usually restricted to urban areas with a large number of observations covering the entire surface (such as Washington D.C [37, 40] or Pittsburgh [49]). Our case is rather the opposite, having a small number of observations concentrated geographically around specific points (see Fig 1).

The existence of these clusters in the data is a problem in most standard spatio-temporal methods, e.g., the Knox-test [37] would always reject the hypothesis of independence under these conditions due to the geographical concentration. Therefore, we can only consider the temporal distribution of femicides. Additionally, the objective of spatio-temporal models is the quantification of the temporal and spatial window of contagion. However, quantification of an effect should be carried out when there is significant proof of its existence (which is not our case as explained in the results section). For these reasons, the Poisson process has been chosen as the main methodology to assess our second research question regarding the existence of a copycat effect in femicides.

#### Poisson process

The Poisson point process, or Poisson process, is usually used as a counting process for rare events. This stochastic process has several very convenient mathematical properties that have made it widely used in very different fields for more than 100 years [50]. Our interest in this processes relies on the fact that, under this distribution, events are randomly located in time.

**Fig 2:**
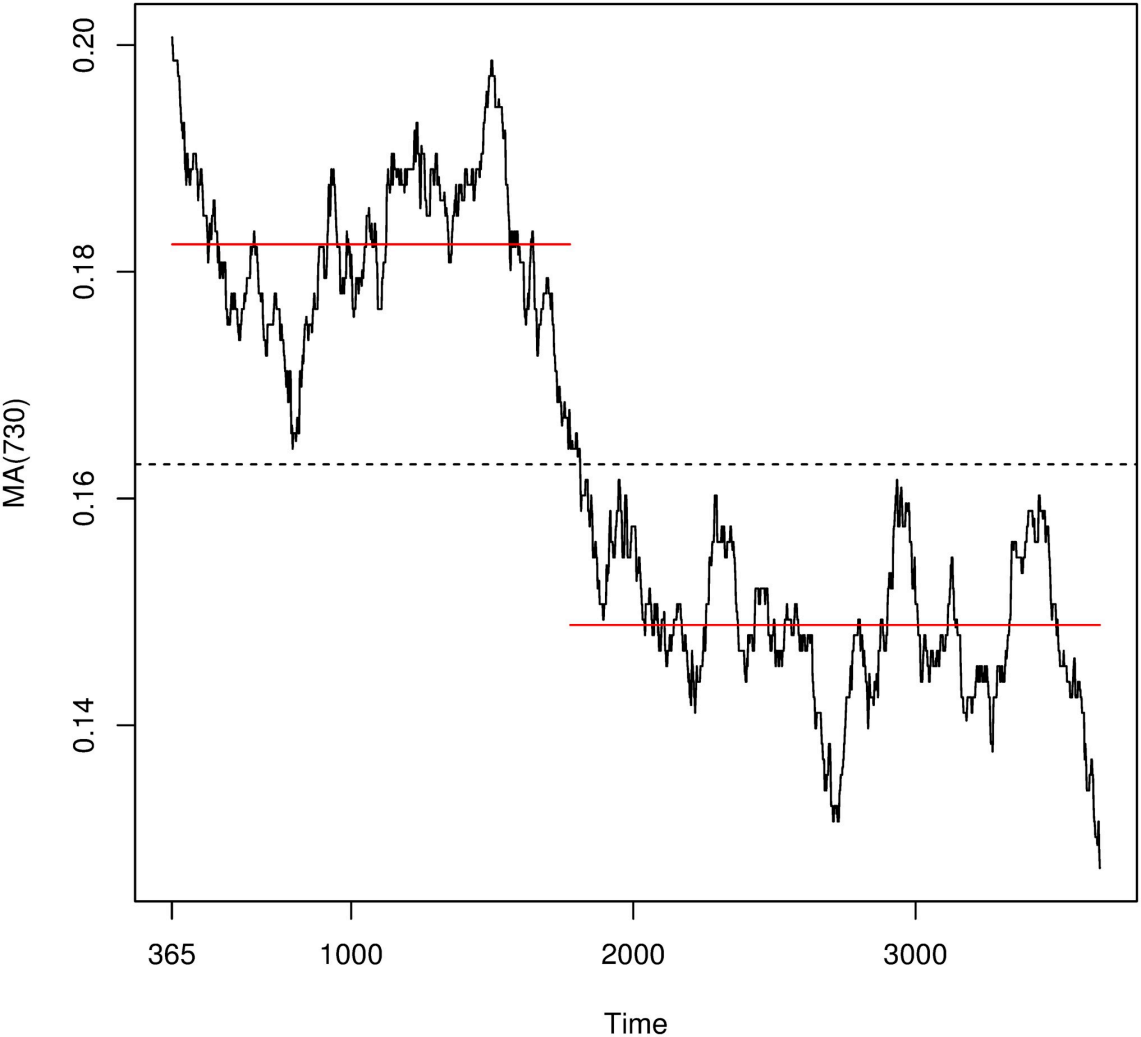
Plot of moving average of order 720 (y-axis) over time (x-axis). The red lines show the average before and after the change-point identified by the analysis. Please notice that the first and the last 365 days are not represented due to the Moving Average.

Let *N*(*t*), *t* ≥ 0 be a Poisson process, in our case *N*(*t*) would be the number of femicides occurred until time *t*. The Poisson process depends on the intensity Λ, which can be interpreted as the ratio of events per unit of time (femicides per day). This, can be a positive constant Λ = λ ≥ 0, or a positive locally integrable function of time, Λ = λ(*t*). In the first case, we have homogeneous or stationary Poisson Processes, and λ can be estimated with the average of the number of events per unit of time. In the second case we talk about non-homogeneous Poisson processes where the intensity Λ depends on the time. The function λ(*t*) can be approximated by local smoothing procedures such as moving averages, as discussed above. As an example, Fig 2 shows the approximation of this function $\hat{\lambda }\left(t\right)$ made with a 2 year window. Different windows generate different estimates but, as seen later, the resulting outcomes are very similar with all considered windows.

In any case, a Poisson process is defined by two main properties: the appearance of the Poisson distribution and the independence between disjoint time intervals [50]. Firstly, for each *t* ≥ 0, the random variables *N*(*t*) follow a Poisson distribution of parameter λ*t* in the homogeneous case and $\int_{0}^{t}\lambda \left(u\right)du$ in the non-homogeneous one. This fits very well many and very different phenomena that happen in reality [51] (some rare diseases, car accidents, bombings, goals, horse kick deaths, etc). In our particular case, we observe a discrete version of the Poisson process since time is discretized by days. Then, if femicides follow an homogeneous Poisson process, the number of daily deaths should fit a Poisson distribution of parameter Λ_*H*_ equals to the average number of femicides per day. On the other hand, if data follows a non-homogeneous Poisson process, *N*(*t*) should fit a Poisson distribution of parameter $\Lambda_{NH}=\int_{0}^{t}\lambda \left(u\right)du$.

Secondly, in a Poisson process disjoint intervals are completely independent, what confers the process the complete randomness property. This means that the occurrence of new events is not affected in any way by what has happened previously. In this way, if the murder series were adjusted to any kind of Poisson process, the existence of a copycat effect would be ruled out since an IPH would not change the probabilities of the following one. We assess the independence of the events using two standard methods: autocorrelation functions and the popular Ljung-Box test (similar to the Box-Pierce test, but with better performance) [52]. The null hypothesis in this test is that data are independently distributed in the sense that correlations are equal to zero.

We also consider the independence between inter-arrival times. The inter-arrival times are denoted as *T*_*k*_ (i.e., the time elapsed in days between the (*k* − 1)-th murder and the next) with 1 ≤ *k* ≤ *N*(*T*), where *T* is the last day considered, *N*(*T*) the final number of events, and *T*_1_ = 0. In both cases we compute the autocorrelation function (ACF), the partial autocorrelation function (PAFC) and perform the Ljung-Box test. Since we are studying daily data, we consider a weekly frequency and run the tests with lag equal to seven and 14 (corresponding to one and two frequency periods). These windows cover all contagion ranges considered previously in the literature on the subject [42, 43].

If the Poisson process is also homogeneous, the events are also identically distributed. In this case, it is known that the process is also characterized by the exponential distribution of the inter-arrival times. Note that this exponential decay of the times between events is a common assumption in contagion models [34].

In our case, the data are limited to discrete times (days). It is easy to verify that the discrete analogue of the exponential distribution of parameter λ is the geometric distribution of parameter *p* = 1 − exp(−λ). To check whether the times *T*_*k*_ are identically distributed with *T*_*k*_ ∼ geom(1 − exp(−λ)) or not, a Chi-square goodness-of-fit test is carried out.

Finally, it is observed that the distribution of events is not uniformly distributed throughout the 11 years under study and there are significant changes in the average and variance of femicides at different time points. Therefore, we must discard the homogeneous Poisson as generator of the data at hand. However, in Fig 2 large plateaus in the intensity function λ(*t*) are also observed (this also happens with other estimations of the intensity function). This would imply that in those regions the process could be locally homogeneous (as a particular case of a non-homogeneous process). This leads us to our final approaches: (a) we split the series into two parts, according to the change-point detected on day 1812, which corresponds to the point where the mean splits the time series in two parts (represented in the figure by a dashed line). So, we denote in advance the first part by *N*_1_(*t*) = *N*(*t*), *t* = 1, 2, …, 1811, and the second part by *N*_2_(*t*) = *N*(*t*) − *N*(1811), *t* = 1812, …, 4018. Then, we check if each of the two parts are drawn from homogeneous Poisson processes. (b) Alternatively, we also consider the whole counting process as drawn from a non-homogeneous Poisson process. If in any of the two approaches positive results were obtained, we could conclude that there are no statistical significant results supporting the existence of a copycat effect in IPHs in Spain.

#### Simulation study

The strength of this study’s conclusions is affected mainly by two factors: i) the relative scarcity of events, that could cause the tests to pass even when there is actually a minimum copycat effect, and ii) the temporal discretization (i.e., daily recorded events), that makes extremely difficult to verify hypotheses about the inter-arrival times and also affects the distribution of the events.

To address these issues and assess the power of our methodology we have carried out an extensive simulation study. The study replicates the previous analysis with hundreds of independent realizations of different stochastic processes (with and without imitation effects) and compares the original series with them, aiming to finding similarities or differences. We consider the following processes:

- Simulated homogeneous Poisson processes ${\hat{N}}_{H}\left(t\right)$ with estimated parameter $\hat{\lambda }$. These processes are defined as:
  $$\begin{array}{c} {\hat{N}}_{H}\left(t\right)={\hat{N}}_{H}\left(t-1\right)+rpois\left(\hat{\lambda }\right), \end{array}$$ (1)
  Where ${\hat{N}}_{H}\left(0\right)=0,\;0\leq t\leq T$. $rpois(\hat{\lambda })$ denotes a randomly generated element from a Poisson distribution of parameter $\hat{\lambda }$. We generate two different processes of this type, ${\hat{N}}_{H,1}\left(t\right)$ and ${\hat{N}}_{H,2}\left(t\right)$ to compare with *N*_1_(*t*) and *N*_2_(*t*) respectively. *T*_1_ = 1811, *T*_2_ = 2207 and the intensity parameters are estimated from the sample: ${\hat{\lambda }}_{1}=N\left(1811\right)/1811≃0.18664$ and ${\hat{\lambda }}_{2}=\left(N\left(4018\right)-N\left(1811\right)\right)/2207\;≃0.14363$.
- Non-homogeneous Poisson processes with intensity function $\hat{\lambda }\left(t\right)$ estimated by moving averages. Since the estimation procedure requires to trim down the extremes of the process to a length equal to the half of the smoothing window *s*, the total length of the simulated processes of this type is 4018 − *s* days. Therefore, we compare them with trimmed original series *N*_*s*_(*t*) = *N*(*t*) − *N*(*s*/2), *t* = *s*/2, …, 4018 − *s*/2. Aiming at covering different scenarios and get more robust conclusions, we have considered *s* ∈ {30, 60, 90, 180, 365, 730} days. Generation of simulated non-homogeneous Poisson processes ${\hat{N}}_{NH}\left(t\right)$ is completely analogous to the homogeneous one:
  $$\begin{array}{c} {\hat{N}}_{NH}\left(t\right)={\hat{N}}_{NH}\left(t-1\right)+rpois\left(\hat{\lambda }\left(t\right)\right) \end{array}$$ (2)
  Where ${\hat{N}}_{NH}\left(0\right)=0,0<=t<=4018-s$ days.
- Processes with copycat effect. We start from a homogeneous Poisson process. The “call-effect” is simulated by an increase in the base intensity λ (the probability of a new murder happening) during the *r* days following a femicide. Previous contributions in the literature consider the increase in femicides due to external factors, see for example [43]. Differently from our model, the authors of [43] consider a multivariate logistic regression model to estimate the increment in the incidence of femicides, while we explicitly increase the event frequency in a Poisson model and compare each realization (obtained through simulation) with our original data.
  Then we have
  $$\begin{array}{c} {\hat{N}}_{\delta ,r}\left(t\right)={\hat{N}}_{\delta ,r}\left(t-1\right)+rpois\left(\hat{\Lambda }\right) \end{array}$$ (3)
  if there have not been femicides in the last *r* days or
  $$\begin{array}{c} {\hat{N}}_{\delta ,r}\left(t\right)={\hat{N}}_{\delta ,r}\left(t-1\right)+rpois\left(\delta \cdot \hat{\Lambda }\right) \end{array}$$ (4)
  otherwise. The parameter *δ* measures the proportion of the increment from the original intensity. In order to cover all possible configurations considered in the related literature [14, 42, 43], we have tested temporal windows, *r*, from 1 to 14 days. In the same way, we have considered increases (*δ*) in the Poisson attribute of 5, 10, 20, 40, 70 and 100 percent, which include the maximum increase in the probability of femicides considered in other papers [42, 43]. To make the processes comparable with our original data, we only keep those simulated series in which the resulting count of events belongs to the 95% confidence interval of $\left(\hat{\lambda }\cdot T\right)$. Aiming at establishing fair comparisons we generate separated processes with copycat from ${\hat{N}}_{H,1}\left(t\right)$ and ${\hat{N}}_{H,2}\left(t\right)$ to be compared with *N*_1_(*t*) and *N*_2_(*t*) respectively. The reason to do this is that, while *N*(*t*) is far from a homogeneous Poisson process, *N*_1_(*t*) and *N*_2_(*t*) are not.

Once these simulated data are generated, similarities between the real data and each of the simulated processes is analyzed by means of homogeneity tests. In particular, we compare: (a) the distribution of the events along time with tests for continuous variables: Kolmogorov-Smirnov and Anderson-Darling; (b) the distribution of the inter-arrival times with the classical Chi-square test of homogeneity. In addition, we have also included a recent test for comparing distributions based on the distance of the characteristic functions called *energy* [53].

The results in next section are averaged over 500 independent runs of each type of process and set of parameters.

## Results

All the tests are interpreted considering a 5% significance level.

### Data exploration

**Fig 3:**
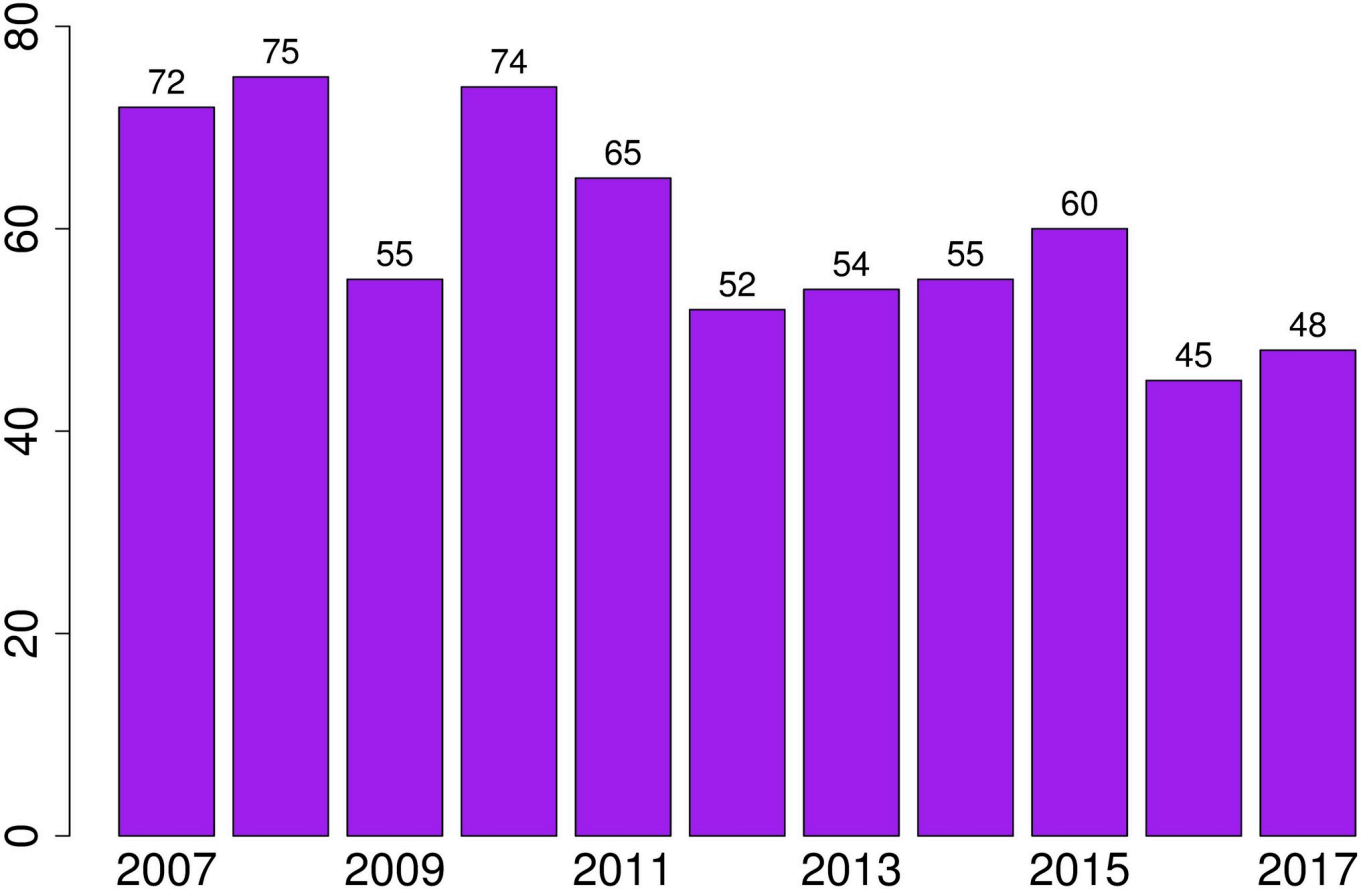
Femicides per year in Spain since 2007.

According to official data (database extraction dated 1st of February 2018), in Spain there have been 655 IPHs from the 1st of January of 2007 to the 31 of December of 2017. The annual distribution can be seen in Fig 3.

In a first analysis, no seasonality or significant differences are observed between different days of the week or months. However, in Fig 3, it seems to appear a slight negative trend in the average number of femicides and a possible outlier in 2009. This would go against the existence of a uniform distribution of IPHs over time as suggested by Teruelo [14].

**Fig 4:**
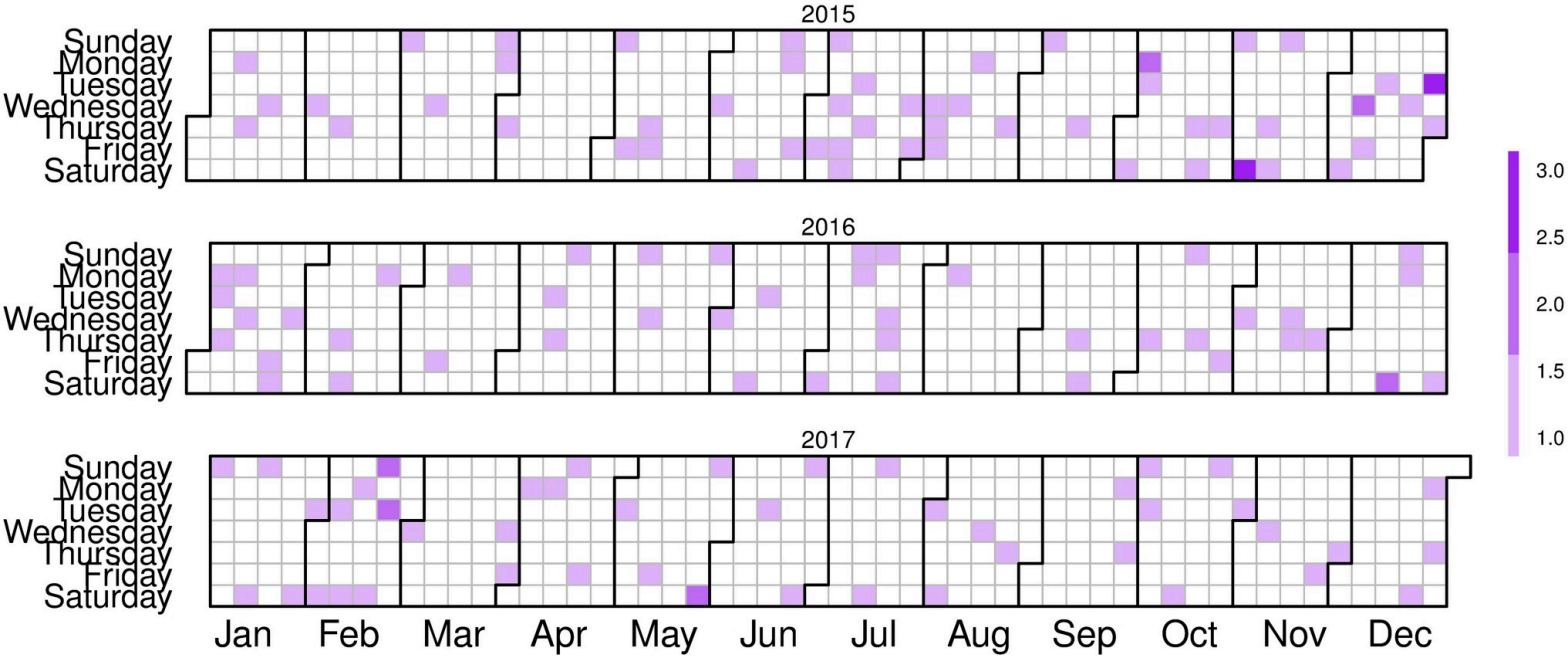
Calendar of femicides in Spain in the last three years.

On the surface, it is difficult to assess whether there is a so-called imitation effect or not. Fig 4 shows the distribution of IPHs along the last three years. Some bouts or concentrations can be clearly appreciated but it can not be ruled out without further testing that they are random effects.

The interested reader could perform further descriptive analysis on the data that are publicly available (at DOI: 10.5281/zenodo.2658915).

### Hypothesis 1: Decreasing trend in the number of femicides

Table 1 shows the p-values of the test concerning the uniform distribution (randomness) of the IPHs along the whole time series.

**Table 1:** Complete series uniformity tests p-values.

Kolmogorov-Smirnov	Cramer von Mises	Anderson-Darling	Runs
0.002400746	0.001173298	0.001138975	0.005094757

All p-values are very small and, therefore, the hypothesis of a random distribution is clearly rejected. As previously noted, some of these tests are known to be very conservative, which gives us even more certainty when rejecting. These results contradict the conclusions in [14] about the uniform distribution of the events.

In addition, Dunn’s test finds that the distribution of IPHs in years 2007, 2008 and 2010 are significantly different to those of 2016 and 2017. The complete results of the Dunn’s test for all pairs of years, trimesters, months and days are in Tables A-D in S1 Appendix. In particular, the number of femicides at the beginning of the time series is significantly bigger than in the last years, indicating a decreasing trend in the number of femicides. No significant difference have been identified between trimesters, months or days of the week.

In the same way, the change-point test with the AMOC criterion over the estimated intensity function $\hat{\lambda }\left(t\right)$ detects a clear change in mean and variance around the end of 2011, independently of the size of the smoothing window used in the estimation of $\hat{\lambda }\left(t\right)$ by moving averages. Table 2 shows the change-points obtained with different windows (from 30 days to two years).

**Table 2:** Change points by moving average order value.

MA window	Change point location (day)
30	1854
60	1862
90	1854
180	1811
365	1728
730	1776

This effect can be observed in Fig 2 where the black curve stands for $\hat{\lambda }\left(t\right)$ estimated with a window of two years and the red lines are the average before and after the change-point. Note that Fig 2 goes from January 1, 2008 to December 31, 2016, as it represents the time series smoothed by a MA with a two-year window. The figure shows a marked difference in the evolution of the intensity function before and after the change-point. In particular, the right side presents a much lower value of intensity, as expected in the presence of a decreasing trend. This decreasing trend is confirmed by the Cox-Stuart test where *H*_1_ is the existence of a decreasing trend. All p-values obtained for different estimations of $\hat{\lambda }\left(t\right)$ are smaller than 10^−25^.

It is important to notice that the change-point was not chosen for a particular reason, it has been identified by the statistical analysis, and the authors were not aware of its existence beforehand. The identification of the reasons behind the change of behavior fall out of the scope of this research and should be the subject of a future study.

### Hypothesis 2: There is no copycat effect in femicides

In the following, we show the results of the tests described in the “Modeling and methods” section to verify if *N*_1_(*t*) and *N*_2_(*t*) resemble a homogeneous Poisson process. Let us recall that the whole series of femicides *N*(*t*) is divided in *N*_1_(*t*) and *N*_2_(*t*), as explained previously, by the change-point at 1811 (since all change-points are close we took and intermediate value, see Table 2 for details).

**Fig 5:**
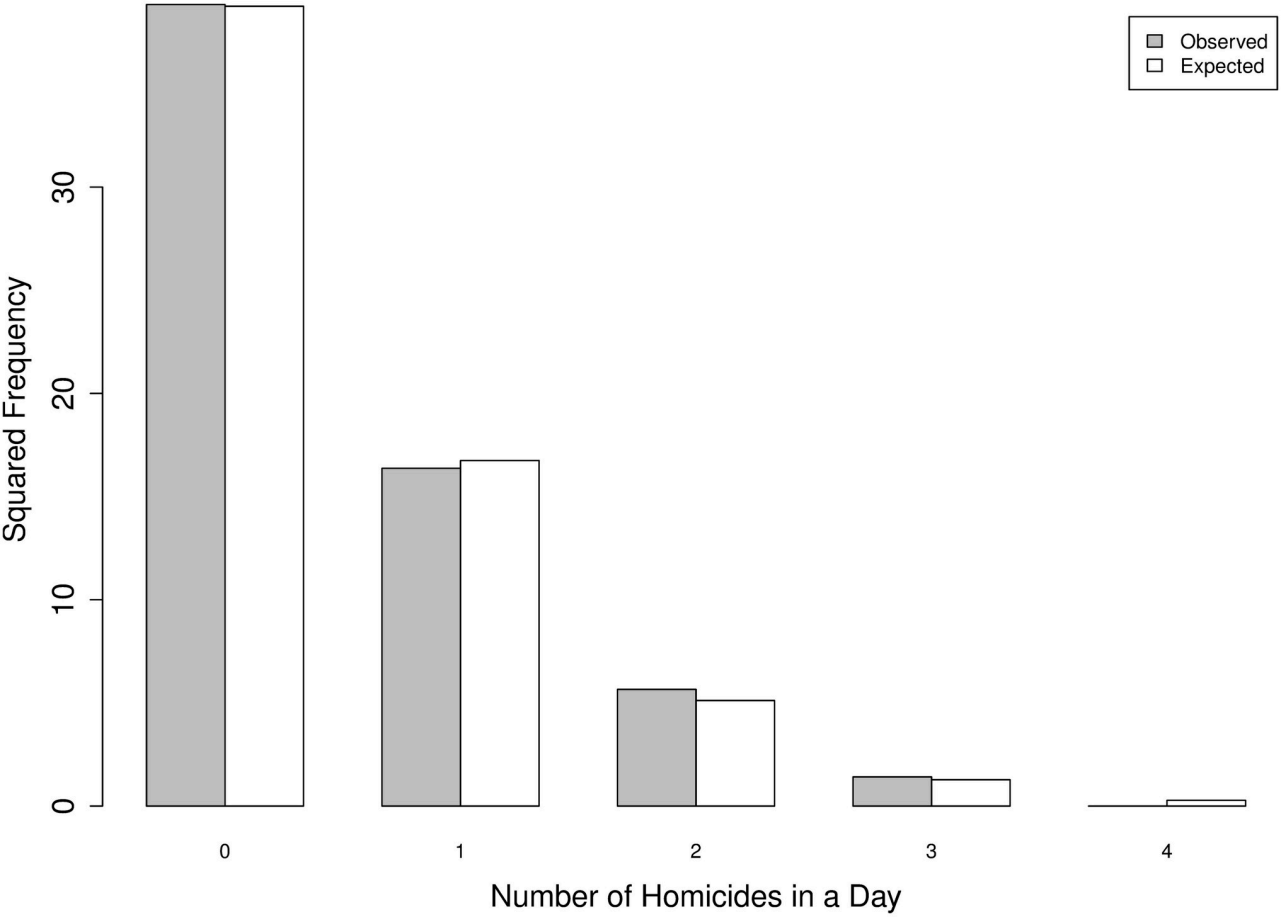
IPHs per day: Square root of frequencies observed (gray) and expected (white) from a Poisson process.

**Fig 6:**
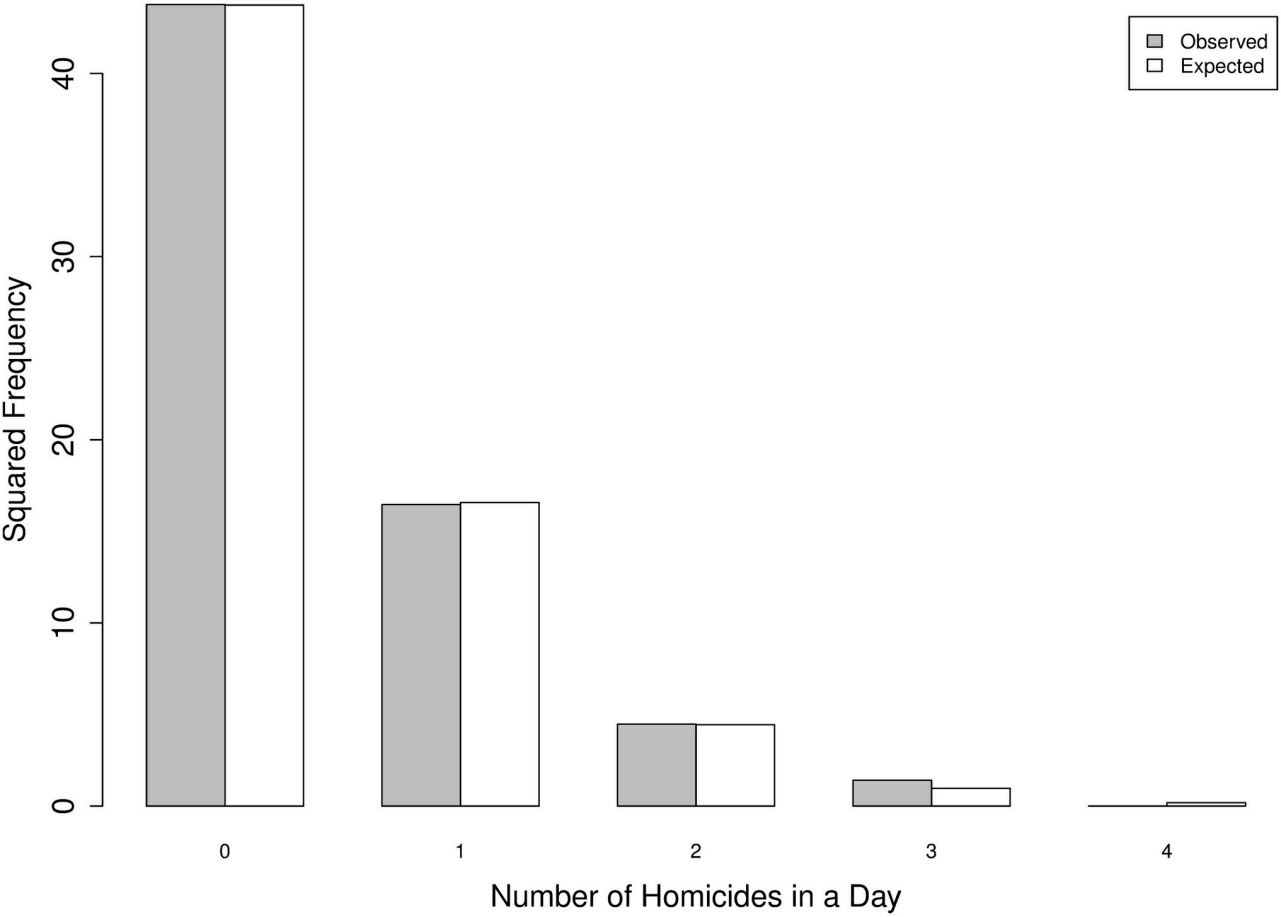
IPHs per day: Square root of frequencies observed from *N*_1_(*t*) (gray) and expected (white) from a Poisson process.

Let us consider a Poisson distribution characterized by a parameter $\hat{\lambda }$ equal to the corresponding sample mean (the maximum-likelihood estimator), in our case $\hat{\lambda }=655/4018≃0.1630$. A comparison of the observed and expected frequencies for *N*_1_(*t*) and *N*_2_(*t*) are given in Table 3 and are visually represented in Figs 5 and 6.

**Table 3:** IPHs per day in *N*_1_(*t*) and *N*_2_(*t*): Frequencies observed and expected from a Poisson process.

#	Observed *N*_1_(*t*)	Expected *N*_1_(*t*)	Observed *N*_2_(*t*)	Expected *N*_2_(*t*)
0	1509	1502.67	1915	1912.7
1	268	280.45	271	274.6
2	32	26.17	20	19.71
3	2	1.63	2	0.94
4	0	0.08	0	0.03

From both the tables and figures, a high degree of similarity between the observed and expected frequencies can be detected. Next, we continue the analysis by testing some specific properties.

#### Properties of a homogeneous Poisson process

As explained in the “Modeling and methods” section, homogeneous Poisson process have specific properties that would be found in case our data corresponded to a process of this type. Table 4 shows the p-values corresponding to different tests (columns) that assume as null hypothesis the fulfillment of these different properties. The tests are applied to the complete series and the two sub-series *N*_1_(*t*) and *N*_2_(*t*) (rows). From left to right: *χ*^2^ of goodness-of-fit for the Poisson distribution (*χ*^2^ − Poiss), over-dispersion/under-dispersion test for the Poisson distribution (Poiss-disp), *χ*^2^ of goodness-of-fit for the geometric distribution for the inter-arrival times (*χ*^2^ − times), Kolmogorov-Smirnov (KS), Cramer von Mises (CVM), Anderson-Darling (AD) and a run test (runs). Reference test parameters, if needed, are estimated by maximum-likelihood. In the table, significant differences are identified with an asterisk (*).

**Table 4:** p-values of different test for *N*(*t*), *N*_1_(*t*) and *N*_2_(*t*) processes. Significant differences (*α* = 0.05) are identified with an asterisk (*).

Process	*χ*^2^ − Poiss	Poiss-disp	*χ*^2^ − Geom-times	KS	CVM	AD	runs
*N*(*t*)	0.5372	0.1803	0.0024*	0.0024*	0.0012*	0.0011*	0.0051*
*N*_1_(*t*)	0.6077	0.2644	0.0325*	0.8553	0.8173	0.8142	0.0037*
*N*_2_(*t*)	0.7961	0.5583	0.1354	0.3855	0.5695	0.5811	0.4118

While most hypotheses are clearly rejected for the whole process *N*(*t*), the situation is quite the opposite for the two sub-series. For *N*_2_(*t*) we cannot reject none of the hypothesis, so, in terms of these characteristics, there is no evidence against *N*_2_(*t*) coming from an homogeneous Poisson process. On the other hand, *N*_1_(*t*) is closer to a homogeneous process than *N*(*t*) but fails two test: distribution of the inter-arrival times (that, on the other hand, would be passed with a significance level *α* = 0.01) and runs. However, these results could be a consequence of using daily data. In fact, the hourly difference between two events having a daily difference of one, ranges between one hour (i.e., the first happening at the end of a day and the next one happening at the beginning of the next day) and 47 hours (i.e., the first happening at the beginning of the day and the next one happening at the end of the next day). This means that two events could have a difference of just a few hours and, therefore, no copycat effect could take place, but still be assigned to different days. As the mass of probability is concentrated mostly in the first two days (corresponding to a difference of zero and one day), this effect biases the results of the Chi-square goodness-of-fit test. Therefore, to moderate this effect, we joined the first two days into a single category, obtaining p-values 0.0224, 0.1244 and 0.2624 for *N*(*t*), *N*_1_(*t*) and *N*_2_(*t*), respectively. As a consequence, according to these tests, inter-arrival times are not drawn from a geometric distribution for the whole process *N*(*t*), while the tests accept that they are drawn from a geometric distribution for *N*_1_(*t*) and *N*_2_(*t*). The failure of the runs test (see last column of Table 4) could indicate some unusual grouping or gaps in this period. A more detailed study year by year suggests that this “non-uniform” behavior is specially acute in 2011 where runs and AD test provides specially low p-values (this study is not included in the paper for the sake of readability, but available in data shared at DOI: 10.5281/zenodo.2658915). Further research is required to ascertain the cause of this result.

#### Independent events

**Fig 7:**
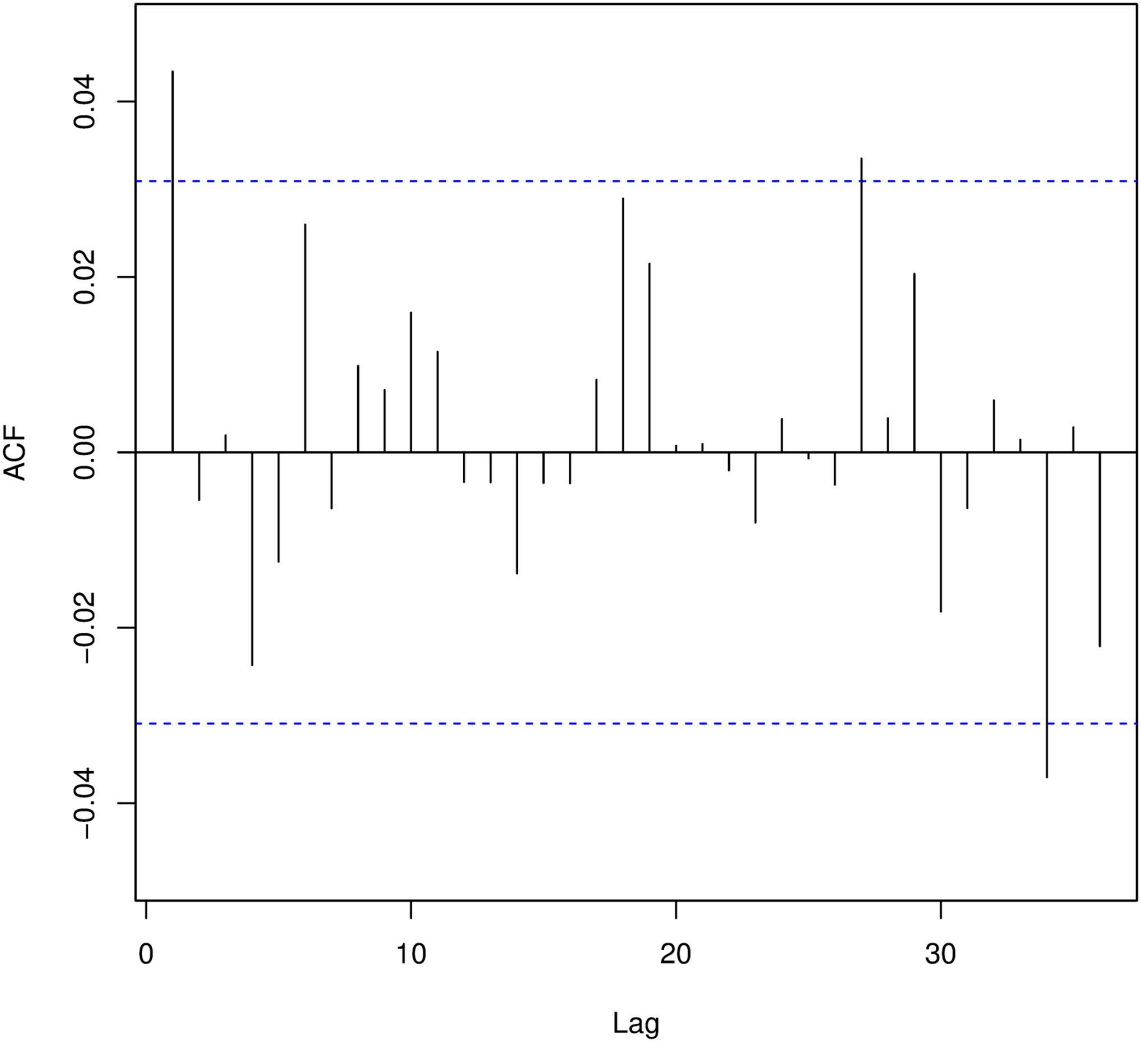
Autocorrelation function of events.

**Fig 8:**
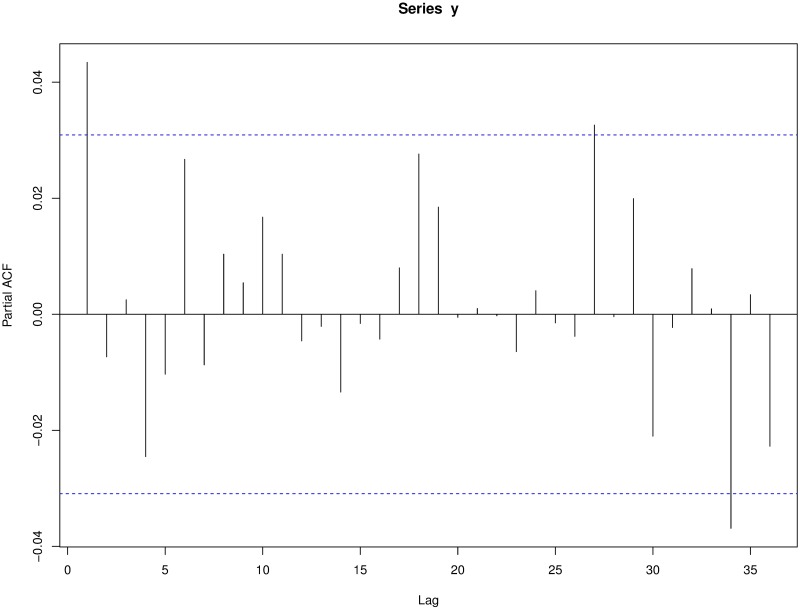
Partial autocorrelation function of events.

Neither the autocorrelation function (Fig 7) nor the partial autocorrelation function (Fig 8) show evidence of correlation among the events (analogous results are obtained when considering *N*_1_(*t*) and *N*_2_(*t*) instead of *N*(*t*) or the inter-arrival times instead of the events).

Only a few lags have statistically significant autocorrelations, albeit some negative. This result could be due to a stochastic effect. Therefore, we run a *Ljung-Box* test setting the number of lags to 7 and 14 to test the data for independence. The results of these tests for *N*(*t*), *N*_1_(*t*) and *N*_2_(*t*) are shown in Table 5.

**Table 5:** P-values of Ljung-Box test applied to events and inter-arrival times of *N*(*t*), *N*_1_(*t*) and *N*_2_(*t*). Significant differences (*α* = 0.05) are identified with an asterisk (*).

Lags	Events			Times		
	*N*(*t*)	*N*_1_(*t*)	*N*_2_(*t*)	*N*(*t*)	*N*_1_(*t*)	*N*_2_(*t*)
7	0.0592	0.0823	0.2463	0.5868	0.6349	0.5790
14	0.2789	0.4842	0.3901	0.4152	0.6273	0.0189*

**Fig 9:**
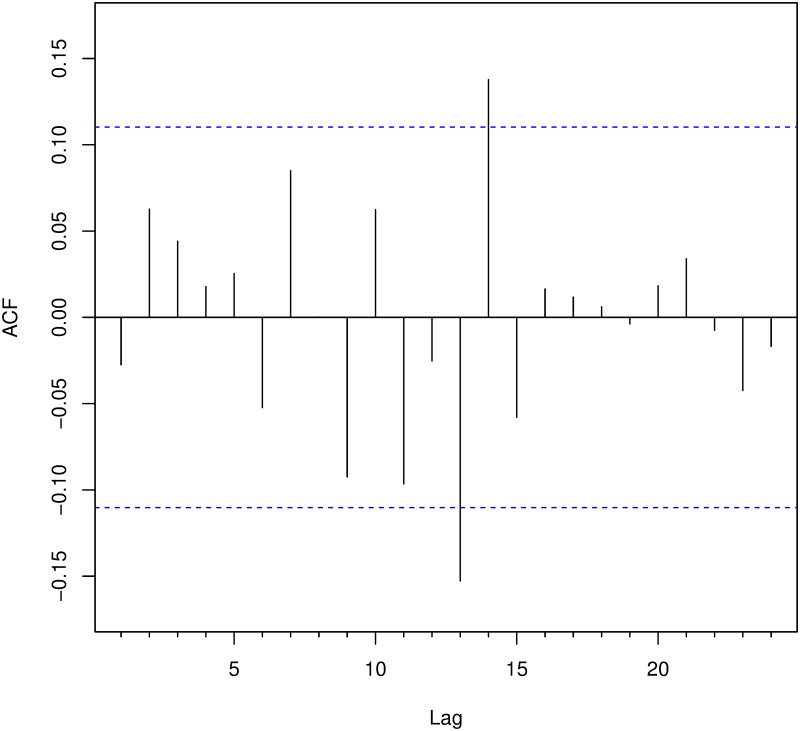
Autocorrelation function of inter-arrival times.

According to Table 5, the series of the events do not show significant relations. In fact, the Ljung-Box tests accepts the hypothesis of independence of the events in both periods. However, when looking at the time data, the series *N*_2_(*t*) is found to be not independent for lag 14. Fig 9 illustrates the ACF for the time data of the time period *N*_2_(*t*).

It can be appreciated that two autocorrelations are significant, at lags 13 and 14. In particular, the one at lag 13 is negative, which would represent a dissuasive effect that is incompatible with the existence of a copycat effect. Further Ljung-Box tests with larger lags accept the null hypothesis of independence (p-value >> 0.05). Therefore, the low p-value for lag equal to 14 seems to be due to spurious correlations.

In summary, the results of the autocorrelation analysis do not allow us to reject the null hypothesis of independence. To complement these results, we perform an extensive simulation study with the double objective of seeing how well-known processes react under the same tests and of finding direct relations between these models and the real data.

#### Simulation comparison

After generating the simulated data as detailed in the simulation study section, we proceed to assess their properties and their similarity with our dataset. Results are summarized in Tables 6, 7 and 8.

**Table 6:** Medians of 500 p-values of different test for the simulated processes. Significant differences (*α* = 0.05) are identified with an asterisk (*).

Process	*χ*^2^ − Poiss	Poiss-disp	*χ*^2^ − Geom-times	KS	CVM	AD	runs
*P*_*H*_	0.6757	0.4529	0.0095*	0.4886	0.4912	0.3806	0.5154
$P_{H_{1}}$	0.6744	0.4899	0.0220*	0.4814	0.4799	0.3661	0.5272
$P_{H_{2}}$	0.7349	0.4835	0.0425*	0.4957	0.4596	0.4045	0.5367
*P*_*NH*_	0.6062	0.4605	0.0044*	0.0022*	0.0021*	0.0011*	0.4179
*PC*_1,0.05_	0.6880	0.4887	0.0229*	0.4977	0.4893	0.3853	0.4908
*PC*_1,0.1_	0.6940	0.4897	0.0182*	0.4815	0.4726	0.3664	0.4875
*PC*_1,0.2_	0.6842	0.4894	0.0115*	0.4363	0.4388	0.3452	0.4491
*PC*_1,0.4_	0.6733	0.4871	0.0047*	0.3862	0.4002	0.2868	0.3938
*PC*_1,0.7_	0.6755	0.4986	0.0015*	0.3175	0.3460	0.2367	0.2506
*PC*_1,1_	0.6555	0.4729	0.0010*	0.2632	0.3012	0.1866	0.1433
*PC*_2,0.05_	0.7229	0.4938	0.0425*	0.4926	0.4863	0.4142	0.4725
*PC*_2,0.1_	0.7211	0.4857	0.0334*	0.4610	0.4587	0.3776	0.4735
*PC*_2,0.2_	0.7286	0.4868	0.0191*	0.4417	0.4381	0.3585	0.4598
*PC*_2,0.4_	0.7126	0.4792	0.0075*	0.3731	0.3833	0.3111	0.3459
*PC*_2,0.7_	0.6943	0.4844	0.0017*	0.2977	0.3361	0.2572	0.1976
*PC*_2,1_	0.6817	0.4707	0.0010*	0.2422	0.2736	0.1868	0.0745

**Table 7:** Medians of 500 p-values of Ljung-Box test applied to the events of the simulated processes for different models (columns). Significant differences (*α* = 0.05) are identified with an asterisk (*).

Lags	*P*_*H*_	$P_{H_{1}}$	$P_{H_{2}}$	*P*_*NH*_	*PC*_1,0.05_	*PC*_1,0.1_	*PC*_1,0.2_	*PC*_1,0.4_	*PC*_1,0.7_	*PC*_1,1_	*PC*_2,0.05_	*PC*_2,0.1_	*PC*_2,0.2_	*PC*_2,0.4_	*PC*_2,0.7_	*PC*_2,1_
7	0.5041	0.4771	0.5264	0.2459	0.5039	0.4857	0.4042	0.2058	0.0424*	0.0095*	0.4967	0.4623	0.3908	0.1612	0.0158*	0.0009*
14	0.5066	0.4910	0.5272	0.1848	0.4929	0.4846	0.4252	0.2403	0.0693	0.0152*	0.4980	0.4669	0.4152	0.1963	0.0299*	0.0018*

**Table 8:** Median p-values over 500 simulations for different homogeneity tests over the distribution of events and inter-arrival times. Significant differences (*α* = 0.05) are identified with an asterisk (*).

Comparison	KS-events	AD-events	Energy-events	*χ*^2^-times	Energy-times
*N*(*t*) vs *P*_*H*_	0.0408*	0.0229*	0.0299*	0.3387	0.3781
*N*_1_(*t*) vs $P_{H_{1}}$	0.6487	0.6493	0.6269	0.2752	0.4776
*N*_2_(*t*) vs $P_{H_{2}}$	0.5498	0.5828	0.5771	0.6453	0.5721
*N*(*t*) vs *P*_*NH*_	0.8663	0.8469	0.8209	0.3897	0.4540
*N*_1_(*t*) vs *PC*_1,0.05_	0.6548	0.6514	0.6430	0.3050	0.4714
*N*_1_(*t*) vs *PC*_1,0.1_	0.6352	0.6275	0.6281	0.2899	0.4764
*N*_1_(*t*) vs *PC*_1,0.2_	0.6143	0.6150	0.6070	0.2920	0.4701
*N*_1_(*t*) vs *PC*_1,0.4_	0.5727	0.5668	0.5684	0.2167	0.4167
*N*_1_(*t*) vs *PC*_1,0.7_	0.5284	0.5294	0.5249	0.1135	0.3085
*N*_1_(*t*) vs *PC*_1,1_	0.4894	0.4747	0.4876	0.0551	0.1741
*N*_2_(*t*) vs *PC*_2,0.05_	0.5296	0.5579	0.5510	0.6342	0.5684
*N*_2_(*t*) vs *PC*_2,0.1_	0.5263	0.5549	0.5460	0.6502	0.5970
*N*_2_(*t*) vs *PC*_2,0.2_	0.4906	0.5122	0.5149	0.6061	0.6045
*N*_2_(*t*) vs *PC*_2,0.4_	0.4801	0.5304	0.5191	0.6089	0.6078
*N*_2_(*t*) vs *PC*_2,0.7_	0.4339	0.4384	0.4552	0.3610	0.2600
*N*_2_(*t*) vs *PC*_2,1_	0.3860	0.4071	0.4167	0.2214	0.1206

Tables 6 and 7 follow the same structure as Tables 4 and 5 respectively. Table 8 presents straightforward comparisons between our data and the simulated models. Different comparisons are in rows and columns stand for different homogeneity tests (KS and AD). The results are the median of the p-values over 500 independent runs. Because of the wide variety of simulated processes, the results of the non-homogeneous Poisson models are aggregated in tables. Likewise, the results of the processes with copycat effect are aggregated by the intensity of the effect *δ*. In the tables we use the following notation to refer to the simulated processes. *P*_*H*_, $P_{H_{1}}$ and $P_{H_{2}}$ stand for homogeneous Poisson processes with the length and parameter $\hat{\lambda }$ corresponding to *N*(*t*), *N*_1_(*t*) and *N*_2_(*t*) respectively, generated using Eq 1. *P*_*NH*_ denotes the aggregation of non-homogeneous Poisson processes with different smoothing parameters, generated using Eq 2. *PC*_1,*δ*_ and *PC*_2,*δ*_ refer to the aggregation of processes with copycat effect with parameter *δ* which are based on $P_{H_{1}}$ and $P_{H_{2}}$ respectively, generated using both Eqs 3 and 4. The complete results without aggregation are available at DOI: 10.5281/zenodo.2658915.

In both Tables 6 and 7, none of the simulated processes replicates perfectly the results obtained with the real data. However, results for *N*_1_(*t*) and, in particular, *N*_2_(*t*) are much closer to those of the homogeneous Poisson processes. Dissimilarities with copycat processes are clear, in particular for higher values of *δ* when comparing the distribution of times, uniformity and, above all, the independence of the events where the Ljung-Box test is frequently rejected for these simulations (in the disaggregated data, the hypotheses of uniformity and uncorrelation are rejected 491 times out of 500 in the copycat processes). In addition, Table 6 shows the problems incurred when using a daily discretization of time, since the hypothesis that the inter-arrival times of the events follow a geometric distribution is rejected even with real Poisson processes (preliminary experiments, not included for the sake of length and readability, show that this effect is corrected when the events are generated by hours).

Table 8 illustrates a similar scenario. Results are not conclusive—except for rejecting that *N*(*t*) comes from an homogeneous Poisson process—but there is bigger coincidence (i.e., larger p-values) between the data and the processes without a copycat effect, especially the non-homogeneous Poisson process. Please note that the results regarding the non-homogeneous Poisson process have been calculated as the median over the results obtained with all the values of the smoothing parameter *s* (see Eq 2), as the results obtained with different values of *s* are quite similar. In the disaggregated data, the hypothesis of homogeneity between *N*_1_(*t*) and *N*_2_(*t*) with the corresponding copycat alternatives is directly rejected in a few cases (5 times with *α* = 0.05 and 33 with *α* = 0.1).

## Discussion

When considering the complete series of femicides, it is shown that the distribution of murders is not uniform over time (in contrast to the conclusions of [14]). In particular, there is a significant decreasing trend and there are strong indications of an important change in the distribution of this type of events at the end of 2011. The causes of these changes in the distribution of femicides are unknown and further research is required to understand this behavior and to continue reducing the incidence of this crime.

When splitting the historical series in two parts according to a change-point, different behaviors can be observed. Roughly speaking, from 2007 to 2011, the femicides can be assumed independent in terms of autocorrelation. On the other hand, statistically significant autocorrelations are detected in the inter-arrival times from 2012 to 2017. However it is not possible to determine if this is due to a copycat effect or to other causes, partly because one of the significant autocorrelation is negative, which would represent a dissuasive effect, opposite to the existence of a copycat effect.

The simulation study reinforces these conclusions. The data are more similar to the processes without copycat effect, Also, the processes with copycat effect show structural differences with respect to the historical series (clearly rejecting many of the tests proposed). However, the differences appear mainly for larger increases in the probability of a new murder. In fact, under this simple model, only increments in the probability of occurrence higher than a 40% can be clearly detected. Therefore, increments like those proposed in part of the literature [42, 43], which are higher than this threshold, can be discarded. These differences in terms of results could be due to the amount of data analyzed, to the quality of the data used, or to the time periods considered. In particular, as explained, there was a behavioral change in 2011 and, therefore, there could have been another one in previous years.

## Conclusions

The statistical analysis undertaken allows for several conclusions, presented in the following: i) The average number of murders is decreasing. We leave for a future study the identification of the causal factors so as to identify effective measures. ii) IPHs occur with a known average rate and independently of the time passed since the last event. Therefore, no copycat effect is observed. This means that, given one femicide, the probability of another IPH occurring in the following days (up to 15 days) is not affected by this event. Also, on average 59.54 femicides per year are expected, regardless of their time distribution that is random. In summary, the tests show no proof of the existence of a copycat effect, especially from 2012 when the data conforms to a homogeneous model. However, it is important to notice that our methodology is not capable of detecting a small copycat effect.

These insights suggest that the occurrence of previous femicides is not a useful predictor for IPH forecasting on a national scale. This implies that future research must continue to look for possible patterns and factors that help predict and prevent IPHs, disregarding the temporal correlation between events. Also, the analysis cannot be used to provide an operational forecast of the occurrence of IPHs. In fact, the results only allow to predict 0.1648 IPHs per day, on average.

It is important to note that the methodology assumes that the authors of the IPHs were aware of previous events on the day of the homicide. According to official sources, all IPHs in Spain are given extensive media coverage on the same day and the days after the event (see, for example, [54]). Although realistic, this might not necessarily be true. The interaction between previous IPHs and the level of exposure of the perpetrators to those events might prove to be significant in an explanatory model of femicides. Most of the limitations of these investigations derive from their low ecological validity, being unable to control and know with certainty that the subjects were exposed to the media. However, in our case this exposure has been contrasted. As part of the “on the field” revision of Spanish femicides that has been carried in the Ministry of Interior project “Detailed review of women intimate partner homicides in Spain” [55], incarcerated murderers have been interviewed by expert psychologists. One of the many questions asked is if the fact of having seen in the media the recent news about similar murders had any influence on the crime. In the almost 100 interviews that have already taken place, none answered affirmatively. And, in fact, this question usually amazed the interviewees which affirmed that they did not take the news into account. This fact is consistent with the way of killing and subsequent reactions: most of the killings had no prior planning; 22% of the murderers committed suicide afterwards, 13.3% tried to commit suicide, 22.7% fled and tried to hide without success, and the remaining 42% surrendered without opposing any resistance.

Finally, future researches could investigate predictive factors of femicides, such as covariates related to the territorial distribution (e.g., educational level, socioeconomic indicators), the perpetrators and the victims.

### Limits

The main limitation of this analysis lies in the local nature of the dataset. In principle, the conclusions drawn can be applied exclusively to Spain. It cannot be excluded *a priori* that other countries experience a copycat effect in femicides. The methodology proposed can be applied to local dataset from other countries to ascertain the existence of this phenomenon. However, although we have focused on the Spanish case, cross-national similarities between different countries shown in studies such as Weldon [56] suggest that there could be a general trend. In particular, organizations such as WHO study cases of violence against women and femicides by dividing the world into regions by income and assuming a certain homogeneity in these regions [57]. According to that, Spain is grouped with other western European countries, Australia, Canada, Japan, South Korea, Iceland, Israel, New Zealand and the United States of America. Therefore, the results of this study could be extended to these countries. However, it would be convenient to repeat the study with data from each country to confirm the results, or to study the possible deviations of the Poisson model according to the different societies, as other researches have indeed found cultural differences in aggressive behavior [58].

### Sociological implications

As stated in the introduction, multiple occurrences of IPHs in a short period of time raise alarms in the population and NGOs concerned with IPV, urging local and national governments to react and adopt preventive measures. The analysis’ results show that clusters of events can occur, although no temporal correlation exists between them. This research informs policy-makers and activists that prevention activities against femicides, though necessary, should be carefully planned and not be the result of a reaction to an apparent increase in the ratio of IPHs. Instead, these activities should be focused to counter real determining factor (e.g., neighborhood environment [59], lifestyle [60], background and previous relationships [61]) to maximize their impact and efficacy and reducing IPHs as much as possible. Also, the lack of a temporal correlation between IPHs indicates that the current media news coverage has no effect and, therefore, nothing suggests that there is a need for a specific journalism regulation.

Future work on the subject should focus on a detailed review of femicides on the field, looking for explanatory reasons. In Spain, since the end of 2015, the “Equipo nacional de revisión pormenorizada de homicidios en el contexto de la violencia de género” (National team for a detailed review of homicides in the context of gender violence) (González et al., 2017) is trying to find the precise explanation for each violent death, shedding some light on the risk factors that are detected. This would allow to refine risk assessment models in the VioGén System by incorporating a scale of mortal risk to prevent this type of events and, thus, warn potential victims. Another important aspect, that has not been fathomed so far, is the relationship between IPVs and IPHs. In fact, research should understand to which extent an IPV results in an IPH. In case that, as expected, the latter derives from the former, educating on social conscience and to recognize signs of IPV [62] becomes even more important, as it would result in increasing the number of IPV reports (note that according to official data from 2007 to 2017 in Spain only 16.75% of the murderers had been reported for IPV by their victim), thus improving the efficacy of the available measures for assistance and control, such as the VioGén system.

In conclusion, femicides are still an open topic that needs investigation. The contribution of this paper is stating with certainty that further preventive actions should not focus on their temporal relations and that there is no evidence of a copycat effect. Hence, news and media coverage reporting of these crimes should not be prevented.

## Supporting information

S1 Appendix Dunn’s test distribution analysis tables. This appendix contains Tables A-D.
(PDF)
