# Correction: Evolution and study of a copycat effect in intimate partner homicides: A lesson from Spanish femicides

**DOI:** 10.1371/journal.pone.0224840

**Published:** 2019-10-30

**Authors:** José L. Torrecilla, Lara Quijano-Sánchez, Federico Liberatore, Juan J. López-Ossorio, José L. González-Álvarez

The following information is missing from the Funding statement: The work by Quijano-Sanchez has been supported by the Spanish Ministry of Science and Innovation grant FJCI-2016-28855.
