# Supplementary material for: Evolution and study of a copycat effect in intimate partner homicides: A lesson from Spanish femicides
Source: PLoS One. 2019 Jun 6;14(6):e0217914. doi: 10.1371/journal.pone.0217914 (PMC6553786; doi:10.1371/journal.pone.0217914)
Supplement: S1 Appendix — This appendix contains Tables A-D. (PDF) [file pone.0217914.s001.pdf]

PLoS One Supporting Information - S1 Appendix. Dunn's test distribution analysis tables.

Article title: Evolution and study of a copycat effect in intimate partner homicides: a lesson from Spanish femicides

Authors: José L. Torrecilla, Lara Quijano-Sánchez, Federico Liberatore, Juan J. López-Ossorio, José L. González-Álvarez.

The following Supporting Information is available for this article:

- **S1 Table A. Results of Dunn's test of multiple comparisons on years using rank sums.**
- **S1 Table B. Results of Dunn's test of multiple comparisons on trimesters using rank sums.**
- **S1 Table C. Results of Dunn's test of multiple comparisons on months using rank sums.**
- **S1 Table C. Results of Dunn's test of multiple comparisons on day of the week using rank sums.**

Table A: **Results of Dunn's test of multiple comparisons using rank sums. The columns show: the pair of years considered, the Z statistic and the associated p-value. Significant differences** ($\alpha = 0.05$) **are identified with an asterisk (*).**

Years	Z statistic	Adjusted p-value	Years	Z statistic	Adjusted p-value
2007 - 2008	-0.01	0.49	2013 - 2014	0.07	0.47
2007 - 2009	1.83	0.03*	2007 - 2015	1.35	0.09
2008 - 2009	1.85	0.03*	2008 - 2015	1.36	0.09
2007 - 2010	0.16	0.44	2009 - 2015	-0.49	0.31
2008 - 2010	0.17	0.43	2010 - 2015	1.19	0.12
2009 - 2010	-1.67	0.05	2011 - 2015	0.37	0.36
2007 - 2011	0.98	0.16	2012 - 2015	-0.46	0.32
2008 - 2011	0.99	0.16	2013 - 2015	-0.50	0.31
2009 - 2011	-0.85	0.20	2014 - 2015	-0.57	0.28
2010 - 2011	0.82	0.21	2007 - 2016	2.44	0.01*
2007 - 2012	1.81	0.04*	2008 - 2016	2.45	0.01*
2008 - 2012	1.82	0.03*	2009 - 2016	0.60	0.27
2009 - 2012	-0.03	0.49	2010 - 2016	2.28	0.01*
2010 - 2012	1.65	0.05	2011 - 2016	1.45	0.07
2011 - 2012	0.82	0.20	2012 - 2016	0.63	0.26
2007 - 2013	1.85	0.03*	2013 - 2016	0.59	0.28
2008 - 2013	1.86	0.03*	2014 - 2016	0.52	0.30
2009 - 2013	0.02	0.49	2015 - 2016	1.09	0.14
2010 - 2013	1.69	0.05	2007 - 2017	2.29	0.01*
2011 - 2013	0.87	0.19	2008 - 2017	2.30	0.01*
2012 - 2013	0.04	0.48	2009 - 2017	0.46	0.32
2007 - 2014	1.92	0.03*	2010 - 2017	2.13	0.02*
2008 - 2014	1.93	0.03*	2011 - 2017	1.31	0.10
2009 - 2014	0.09	0.47	2012 - 2017	0.48	0.31
2010 - 2014	1.76	0.04*	2013 - 2017	0.44	0.33
2011 - 2014	0.94	0.17	2014 - 2017	0.37	0.36
2012 - 2014	0.11	0.45	2015 - 2017	0.94	0.17
2013 - 2014	0.07	0.47	2016 - 2017	-0.15	0.44

Table B: **Results of Dunn's test of multiple comparisons using rank sums. The columns show: the pair of trimesters considered, the Z statistic and the associated p-value. Significant differences** ($\alpha = 0.05$) **are identified with an asterisk (*).**

Years	Z statistic	Adjusted p-value
T1 - T2	0.45	0.33
T1 - T3	-0.35	0.36
T2 - T3	-0.80	0.21
T1 - T4	-0.06	0.48
T2 - T4	-0.51	0.30
T3 - T4	0.29	0.39

Table C: **Results of Dunn's test of multiple comparisons using rank sums. The columns show: the pair of months considered, the Z statistic and the associated p-value. Significant differences** ($\alpha = 0.05$) **are identified with an asterisk (*).**

Months	Z statistic	Adjusted p-value	Months	Z statistic	Adjusted p-value
1 - 10	0.03	0.49	3 - 6	-0.60	0.28
1 - 11	0.06	0.48	4 - 6	-0.96	0.17
10 - 11	0.03	0.49	5 - 6	-0.10	0.46
1 - 12	0.59	0.28	1 - 7	-0.64	0.26
10 - 12	0.56	0.29	10 - 7	-0.67	0.25
11 - 12	0.52	0.30	11 - 7	-0.69	0.24
1 - 2	-0.00	0.50	12 - 7	-1.23	0.11
10 - 2	-0.04	0.49	2 - 7	-0.62	0.27
11 - 2	-0.06	0.47	3 - 7	-1.41	0.08
12 - 2	-0.58	0.28	4 - 7	-1.77	0.04
1 - 3	0.77	0.22	5 - 7	-0.91	0.18
10 - 3	0.74	0.23	6 - 7	-0.80	0.21
11 - 3	0.71	0.24	1 - 8	0.55	0.29
12 - 3	0.19	0.43	10 - 8	0.52	0.30
2 - 3	0.76	0.22	11 - 8	0.48	0.31
1 - 4	1.13	0.13	12 - 8	-0.04	0.48
10 - 4	1.10	0.14	2 - 8	0.54	0.30
11 - 4	1.06	0.14	3 - 8	-0.23	0.41
12 - 4	0.55	0.29	4 - 8	-0.59	0.28
2 - 4	1.11	0.13	5 - 8	0.28	0.39
3 - 4	0.37	0.36	6 - 8	0.37	0.35
1 - 5	0.27	0.39	7 - 8	1.18	0.12
10 - 5	0.24	0.41	1 - 9	0.28	0.39
11 - 5	0.21	0.42	10 - 9	0.25	0.40
12 - 5	-0.32	0.38	11 - 9	0.22	0.41
2 - 5	0.27	0.39	12 - 9	-0.30	0.38
3 - 5	-0.50	0.31	2 - 9	0.28	0.39
4 - 5	-0.86	0.19	3 - 9	-0.49	0.31
1 - 6	0.17	0.43	4 - 9	-0.85	0.20
10 - 6	0.14	0.44	5 - 9	0.01	0.50
11 - 6	0.11	0.46	6 - 9	0.11	0.46
12 - 6	-0.41	0.34	7 - 9	0.91	0.18
2 - 6	0.17	0.43	8 - 9	-0.26	0.40

Table D: **Results of Dunn's test of multiple comparisons using rank sums. The columns show: the pair of days of the week considered, the Z statistic and the associated p-value.**

Days	Z statistic	Adjusted p-value
Su - Mo	0.32	0.38
Su - Tu	0.91	0.18
Mo - Tu	0.59	0.28
Su - We	0.99	0.16
Mo - We	0.68	0.25
Tu - We	0.08	0.47
Su - Th	0.54	0.29
Mo - Th	0.22	0.41
Tu - Th	-0.37	0.36
We - Th	-0.45	0.32
Su - Fr	1.90	0.03*
Mo - Fr	1.58	0.06
Tu - Fr	0.99	0.16
We - Fr	0.90	0.18
Th - Fr	1.36	0.09
Su - Sa	0.09	0.46
Mo - Sa	-0.22	0.41
Tu - Sa	-0.82	0.21
We - Sa	-0.90	0.18
Th - Sa	-0.45	0.33
Fr - Sa	-1.81	0.04*
